# Society Meetings

**Published:** 1893-07

**Authors:** John B. Hamilton

**Affiliations:** Executive President


### Society Meetings.

The nineteenth annual meeting of the Mississippi Valley Medical Association will be held in Indianapolis, Wednesday, Thursday, and Friday, October 4, 5, and 6, 1893, under the presidency of Dr. R. Stansbury Sutton, of Pittsburg. The profession of Indianapolis joins in extending a cordial invitation to physicians and their families to attend the meeting. Reduced railroad rates will be provided, and also ample accommodations for the entertainment of the visitors. The Secretary, Dr. Frederick C. Woodburn, will take pleasure in giving any information in connection with the meeting. His address is No. 399 College avenue.

---

THE section on General Surgery of the Pan-American Medical Congress extends a cordial invitation to all medical gentlemen engaged in the practice of surgery, as teachers or practitioners in any of its branches, to participate in all its meetings, and contribute papers for the general information. Such papers should conform to the requirements, as set forth in the general regulations of the Congress.

In view of the wide extent of the constituency of the Congress, and the varied human environment necessarily under observation, it is suggested that the topic of endemic or surgical diseases prevalent in each country might fittingly receive a large share of attention from the members of this section; but carefully written papers upon *any* topic connected with surgical bacteriology, surgical pathology, or operative surgery of the regions, will be welcomed by the section.

JOHN B. HAMILTON,
*Executive President.*

